# Adaptive multi-mode locomotion for bipedal wheel-legged robots via sparse mixture-of-experts deep reinforcement learning

**DOI:** 10.3389/frobt.2026.1788395

**Published:** 2026-02-25

**Authors:** Pan He, Zeang Zhao, Shengyu Duan, Panding Wang, Hongshuai Lei

**Affiliations:** Institute of Advanced Structure Technology, Beijing Institute of Technology, Beijing, China

**Keywords:** bipedal wheel-legged robot, curriculum learning, gradient conflict, mixture of experts, reinforcement learning

## Abstract

The bipedal wheel-legged robot combines the high energy efficiency of wheeled movement with the terrain adaptability of legged locomotion. However, achieving a smooth transition between these two heterogeneous motion modes within a unified control framework remains challenging. This study proposes a reinforcement learning control framework that integrates the Mixture of Experts (MoE) architecture. This approach employs a “divide and conquer” strategy by introducing a dynamic gating network and a Top-K sparse activation mechanism, which automatically allocates different motion modes to specific expert subnetworks, effectively decoupling conflicting gradients. Simulation results demonstrate that, compared to the single-network PPO method, the MoE-enhanced algorithm exhibits significant improvements in training stability and rewards. The learned policy successfully achieved smooth rolling on flat surfaces and transitioned to dynamic leg-lifting gaits when confronted with obstacles. In various test terrains, it showed a markedly higher success rate compared to the single-network PPO method.

## 1 Introduction

The trade-off between wheeled efficiency and legged versatility is a significant issue in mobile robots. While wheeled robots excel on flat terrain and legged robots adapt to unstructured environments, bipedal wheel-legged robots have emerged as a promising hybrid to combine these advantages (Klemm et al., 2019; Wang et al., 2021; Guo et al., 2022; He et al., 2025; Wang et al., 2025). These systems can switch between continuous rolling for cruising and discrete stepping or jumping for obstacles (Klemm et al., 2020; Cui et al., 2021; Lee et al., 2024). However, unlocking this potential requires a control policy capable of mastering these distinct modes simultaneously. Traditional control of wheel-legged robots often relies on model-based optimization. For example, analytical second-order derivatives of rigid-body contact dynamics have been derived to enhance the multi-shooting Differential Dynamic Programming (DDP) algorithm (Singh et al., n.d.), thereby enabling a highly efficient approach to handling complex contact interactions in humanoid and hybrid locomotion robots. While such trajectory optimization methods deliver precise control performance based on analytical gradients, reinforcement learning (RL) provides an alternative by learning robust policies through trial and error, which can be more adaptable to unstructured environments.

Developing a unified control policy is challenging due to the heterogeneous dynamics between high-precision rolling (Bouton et al., 2020; García and Duarte, 2024) and high-frequency maneuvers like jumping (Hwangbo et al., 2019; Lee et al., 2020; Zheng et al., 2025). Attempting to master these diverse skills within a monolithic reinforcement learning agent often triggers severe gradient conflicts (Yu et al., 2020), where updates for one mode interfere with the optimization of another. Consequently, the agent settles for a compromised policy, suffering from catastrophic forgetting or unstable convergence (Kirkpatrick et al., 2017; Schaul et al., 2019).

To address these limitations, this research proposes a Mixture of Experts (MoE) architecture integrated into the PPO framework, which effectively tackles the problem of gradient conflict (Shazeer et al., 2017; Celik et al., 2024; Li et al., 2024; Obando-Ceron et al., 2024; Akrour et al., 2022). The core concept of our approach is “divide-and-conquer.” Instead of forcing a single neural network to encode all locomotion primitives, we employ a dynamic gating mechanism that selects specific expert sub-networks based on the robot’s state. This architecture effectively disentangles the conflicting gradients. One expert can specialize in steady-state rolling, while another focuses on dynamic leg-lifting, with the gating network learning the optimal switching logic. Aside from innovations in algorithm architecture, we also propose a phased curriculum learning strategy (Kumar et al., 2021; Lee et al., 2024). The training process is divided into two phases. In the first phase, the agent is trained on specific terrains, focusing the acquisition of vertical mobility skills. In the second phase, the policy is generalized across randomized mixed terrains. Blind locomotion is realized by relying solely on proprioceptive history without privileged terrain information.

Based on the Diablo bipedal wheel-legged robot platform, we demonstrated the superiority of the proposed MoE-enhanced framework over the single-network PPO method. The results highlight three key contributions:

- Training Stability: A reinforcement learning framework integrated with the Mixture of Experts (MoE) architecture is introduced, which effectively resolves gradient conflicts in multi-modal locomotion tasks, significantly improving training stability and mitigating catastrophic forgetting compared to single-network PPO.
- Higher Performance: A dynamic gating network coupled with a Top-K sparse activation mechanism is introduced. This design automatically decouples heterogeneous motion modes, enabling expert subnet specialization and achieving higher reward peaks for better stability-agility balance.
- Enhanced Traversability: A two-phase curriculum learning strategy is designed to progress from deterministic terrains to noisy complex environments. This approach ensures robust generalization, granting the robot enhanced traversability over unstructured obstacles.

This work offers a novel solution for general motion learning in bipedal wheel-legged robots, providing a scalable pathway for deploying agile hybrid locomotion in complex environments.

## 2 Methods

### 2.1 Overall control framework

**FIGURE 1:**
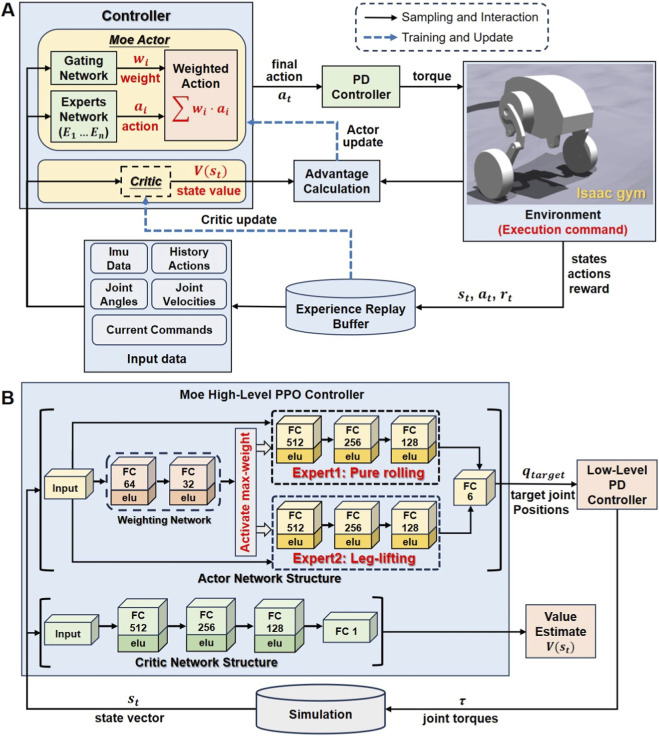
Robot control algorithm framework. **(A)** Overall control framework for the robot training process. **(B)** Composition and principle of the MoE algorithm controller.

The robotic platform utilized in this study is the bipedal wheel-legged robot, Diablo (Direct Drive Tech, China), with simulations conducted in the Isaac Gym environment (Hwangbo et al., 2019; Lee et al., 2020; Kumar et al., 2021; Nahrendra et al., 2023). The overall control framework comprises a high-level decision controller and a low-level PD controller (Figure 1A). The high-level decision controller computes the target positions for each joint of the robot at a given time, which are then be scaled and transmitted to the low-level PD controller. The PD controller calculates the joint torques required to track these target positions, thereby driving the physics simulation.

### 2.2 Design of the controller based on the PPO algorithm

We initially trained the robot using a standard reinforcement learning approach. The objective was to achieve multi-gait capabilities within a single decision-making network. In this section, we employ the PPO algorithm (Schulman et al., 2017) to train the robot, and both the Actor and Critic consist of a neural network with dimension of [512, 256, 128].

#### 2.2.1 Simulation environment setup

The training environment was constructed on the NVIDIA Isaac Gym simulation platform, utilizing an NVIDIA GeForce RTX 3090 Ti graphics card and configured with 4,096 parallel environments to efficiently train the Diablo robot, with a maximum episode duration of 20 s. The environment features specific terrains, which compel the robot to transition from rolling to stepping.

#### 2.2.2 State space

In this study, the state space S is constructed as a high-dimensional composite vector of dimension $R^{153}$ . The goal is to spatialize the temporal information, enabling a standard multi-layer perceptron (MLP) network to effectively perceive dynamic trends in the system, such as changes in acceleration and contact states. This vector is formed by concatenating real-time proprioceptive observations with historical joint data. Crucially, to ensure a truly blind locomotion policy, the input state S relies strictly on onboard proprioceptive sensors (IMU, joint encoders, and command input) and a history sequence of the most recent 10 frames. We explicitly exclude any exteroceptive data or privileged terrain heightmaps from the policy input. This configuration is maintained consistently across both the training and testing phases to validate the robot’s adaptability using only proprioception.

For the proprioceptive observations $O_{base}$ , this component is represented as a vector in $R^{33}$ , reflecting the robot’s current physical state and the task objectives. As shown in Equation 1, the specific elements include: the linear velocity of the robot’s base in the body coordinate system $v_{t}\in R^{3}$ ; the angular velocity $w_{t}\in R^{3}$ ; the projection of the gravity vector onto the body axes $g_{t}^{proj}\in R^{3}$ ; the commands $c_{t}\in R^{6}$ , which encompass the target linear velocities $v_{x}$ , $v_{y}$ and yaw rate $w_{yaw}$ , as well as the jump height target $h_{jump}$ and the adjustment angles for the left and right legs $\theta_{left}$ , $\theta_{right}$ ; the current position error for the joints $e_{t}=\left(q_{t}-q_{default}\right)\in R^{6}$ ; the joint velocities ${\dot{q}}_{t}\in R^{6}$ ; and the action vector from the previous time step $a_{t-1}\in R^{6}$ .

$$O_{base}=<v_{t},w_{t},g_{j}^{proj},c_{t},e_{t},{\dot{q}}_{t},a_{t}>$$ (1)

The historical observations $O_{hist}$ is represented as a vector in $R^{120}$ , which stores the joint data from the most recent 10 frames, corresponding to a continuous segment of time in the physical simulation. This includes the historical position errors of the robot’s six joints $h_{pos}=\left[e_{t-10},...,e_{t-1}\right]\in R^{60}$ and the historical velocities $h_{vel}=\left[{\dot{q}}_{t-10},...,{\dot{q}}_{t-1}\right]\in R^{60}$ . At any given time t , the input state vector is formulated as follows:

$$S_{t}=<O_{base},h_{pos},h_{vel}>$$ (2)

#### 2.2.3 Action space

In this study, we employ a continuous action space $A\in R^{6}$ for the precise control of the six degrees of freedom on both sides of the bipedal wheel-legged robot. The policy network does not directly output torques. Instead, it generates a normalized position deviation vector $a_{t}\in R^{6}$ , which is transformed into target joint positions $q_{t}*$ through linear mapping, as shown in Equation 3. Here, $q_{nom}$ represents the robot’s initial posture, and σ is the action scaling factor. These high-level position commands are then passed to a low-level PD controller to compute the final execution torques $\tau_{t}$ , with the calculation method outlined in Equation 4. In this context, $q_{t}$ and ${\dot{q}}_{t}$ denote the current joint positions and velocities, while $K_{p}$ and $K_{d}$ represent the specified position stiffness and velocity damping gains.

$$q_{t}^{*}=q_{nom}+\sigma \cdot a_{t}$$ (3)

$$\tau_{t}=clip\left(K_{p}\left(q_{t}^{*}-q_{t}\right)-K_{d}{\dot{q}}_{t},-\tau_{\max},\tau_{\max}\right)$$ (4)

#### 2.2.4 Reward function

The reward function R consists of four components: task objective rewards, gait induction and constraint rewards, stability and physical constraint rewards, and motion smoothness and safety rewards. The specific parameters and calculation methods for these reward components are detailed in Table 1. In the gait induction and constraint rewards, it is worth noting that the “leg lifting reward” and “no moonwalk reward” are complementary in their functionality. Specifically, the positive “leg lifting reward” is designed to induce a vertical height difference between the hub centers of the robot’s left and right legs, thereby prompting a leg-lifting and stepping gait while preventing the training process from converging to the local optimum of pure rolling locomotion. In contrast, the negative “no moonwalk reward” is introduced to eliminate horizontal offset between the left and right leg hub centers, effectively suppressing the undesired gait of staggered wheel positions along the forward direction.

**TABLE 1:** Specific reward parameters and calculation methods.

Category	Reward itemname	Scale	Calculationmethod
Task objective rewards	Tracking lin vel	5.5	$r_{lin\_vel}=\exp\left(-{\left\|v_{xy}-v_{xy}^{com}\right\|}^{2}/\sigma_{lin}\right)$
	Tracking ang vel	5.5	$r_{ang\_vel}=\exp\left({-\left(w_{yaw}-w_{yaw}^{com}\right)}^{2}/\sigma_{ang}\right)$
	Base height	−0.25	$r_{height}=-{\left(h_{base}-h_{t\arg et}^{com}\right)}^{2}$
Gait induction and constraint reward	Leg lifting	20	$r_{leg\_lift}=mean\left(\left|z_{wheel-L}-z_{wheel-R}\right|\right)$
	No fly	0.25	$r_{no\_fly}=1\left(if\;\sum contact==2\right)$
	No moonwalk	−2.5	$r_{no\_moonwalk}=-{\left(P_{proj\_L}+P_{proj\_R}\right)}^{2}$
Stability and physical constraint reward	Orientation	−5.5	$r_{orientation}=\left\|g_{xy}^{proj}\right\|$
	Lin vel z	−0.1	$r_{lin\_vel\_z}=v_{z}^{2}$
	Ang vel xy	−0.25	$r_{ang\_vel\_xy}=w_{x}^{2}+w_{y}^{2}$
Motion smoothness and safety reward	Collision	−180	$r_{collision}=-1\left(ifbady\_contact>0.1\right)$
	Dof pos limits	−0.5	$r_{dof\_limit}=-\sum \text{clip}\left(q-q_{\text{limit}}\right)$
	Action rate	−0.008	$r_{action\_rate}=-{\left\|a_{t}-a_{t-1}\right\|}^{2}$
	Torques	-1e-5	$r_{torque}=-{\left\|\tau \right\|}^{2}$

#### 2.2.5 Domain randomization and termination conditions

To enhance the robustness of the policy, this study implements a comprehensive domain randomization framework that includes environmental dynamics randomization, initial state perturbations, sensor noise injection, and external mechanical impacts. The ground friction coefficient μ of the physics engine is independently sampled from a uniform distribution $U\left(0.5,1.25\right)$ during each reset, and the base’s horizontal position is randomly offset within a range of ±1 m. The observation space is augmented with parameterized additive noise, as shown in Equation 5, where $n∼U\left(-s,s\right)$ , and the noise amplitude s is set with differentiated intensities, the specific values are given in Supplementary Table S4 of the Supplementary Material. Furthermore, a random horizontal velocity impact of 1.0 m/s is applied every 15 s to enforce balance recovery. The termination condition $d_{t}$ is triggered by both time truncation and physical contact.

$$O_{noisy}=O_{origin}+n$$ (5)

### 2.3 Design of the MoE controller

Based on the original PPO framework, we introduced a MoE mechanism in the Actor. The algorithm is illustrated in Figure 1B. The new algorithm maintains consistency with the original PPO algorithm in terms of state space, action space, and reward function. For the new algorithm that incorporates the MoE, the Actor is composed of a gating network with dimensions of [128, 64] and two expert networks sized [512, 256, 128]. At each time step, the final action output of the Actor corresponds to the expert network with the highest weight.

#### 2.3.1 Composition of the loss function

The loss function $L_{base}$ of the basic PPO algorithm is shown in Equation 6. Here, $L^{clip}\left(\theta \right)$ denotes the clipped surrogate loss that restricts the magnitude of policy updates, $L^{value}\left(\theta \right)$ represents the squared error loss for evaluating state values, and $H\left[\pi_{\theta }\right]$ is a linear combination of entropy to encourage policy exploration. The coefficients $c_{1}$ and $c_{2}$ are used to adjust the contributions of the latter two losses.

$$L_{base}=L^{clip}\left(\theta \right)+c_{1}\cdot L^{value}\left(\theta \right)-c_{2}\cdot H\left[\pi_{\theta }\right]$$ (6)

After introducing the MoE mechanism, we augmented the total loss function to $L_{total}$ as shown in Equation 7 to prevent mode collapse within the expert networks. The newly added term is the load balancing auxiliary loss $L_{aux}$ , which is regulated by the coefficient $c_{moe}$ . The calculation method for this loss is detailed in Equation 8, where $w_{i}$ denotes the average gating weight of the *i*-th expert in the current training batch, and N represents the total number of experts. This term enforces uniform activation and utilization of all experts by minimizing both the negative entropy of the average weights and the mean absolute deviation.

$$L_{base}=L_{base}+c_{moe}\cdot L_{aux}$$ (7)

$$L_{\text{aux}}=\sum_{i=1}^{N}w_{i}\ln w_{i}+0.5\cdot \frac{1}{N}\sum_{i=1}^{N}\left|w_{i}-\frac{1}{N}\right|$$ (8)

#### 2.3.2 Top-K mechanism in the MoE framework

To achieve computational sparsity and encourage the policy network to transition clearly between different motion modes, we incorporate a Top-K gating mechanism in the policy framework (Chen et al., 2025; D’Souza et al., 2025; Huang et al., 2025; Xu et al., 2025; Vincze et al., 2025). For state input $S_{t}$ , the gating network $G_{\phi }$ generates a probability distribution $g\in R^{N}$ for N experts via Softmax (Equation 9). A truncation operation then retains only the top k experts to form the active set K (Equation 10). Selected weights are re-normalized to ensure effective gradient propagation. The final output $\mu_{t}$ is a linear combination of the outputs $E_{\theta -i}\left(S_{t}\right)$ from these k active experts (Equation 11), enforcing sparse activation and allowing each expert to focus on specific sub-tasks. In this study, the gating network and expert networks take the same input $S_{t}$ (Equation 2). Meanwhile, we fix the number of activated experts K to 1 at each time step, thereby ensuring that only the most relevant expert is active at any given time to maximize the decoupling of motion primitives.

$$g=Softmax\left(W_{g}\cdot s_{t}+b_{g}\right)$$ (9)

$$K=\left\{i|g_{i}\in top-k\left(g\right)\right\}$$ (10)

$$\mu_{t}=\Sigma_{i\in K}w_{i}\cdot E_{\theta_{i}}\left(S_{t}\right)$$ (11)

### 2.4 Phased training design

**FIGURE 2:**
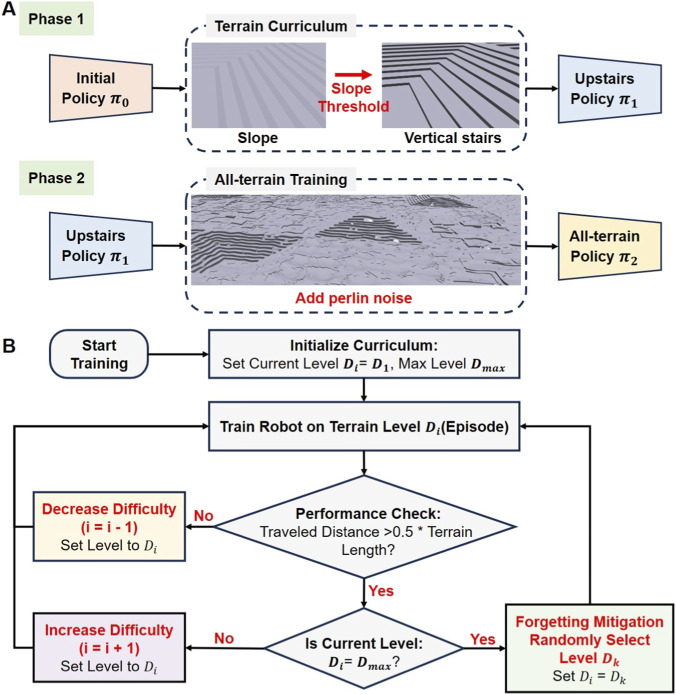
Schematics of the training process. **(A)** Schematic of the phased training design. **(B)** Schematic of the curriculum learning design.

For a bipedal wheel-legged robot, the learning process begins with mastering fundamental equilibrium on planar surfaces, followed by tracking velocity commands via rolling locomotion. Subsequently, the robot negotiates slopes and overcomes vertical obstacles on complex terrains using leg-lifting gaits. Ultimately, it masters dynamically switching between rolling and leg-lifting modes. To this end, we designed a progressive training framework to induce these skills sequentially. Central to this approach is the two-phase training pipeline illustrated in Figure 2A, where each phase features distinct terrain compositions and complexity levels tailored to specific skills. The first phase consists exclusively of flat ground, slopes, and vertical stairs. An inclination threshold is defined such that slopes exceeding this value are rendered as vertical stairs in the simulation. This process evolves the initial policy $\pi_{0}$ into an obstacle-capable policy $\pi_{1}$ . The second phase involves transferring the policy $\pi_{1}$ to a comprehensive terrain set for re-training. To increase terrain complexity, Phase 2 incorporates wave terrain and discrete terrain into the training set, with Perlin noise added to introduce irregular surface perturbations. This phase serves as a comprehensive training ground for hybrid locomotion switching and dynamic balancing. Building upon the results of Phase 1, this process culminates in the final generalized policy $\pi_{2}$ .

### 2.5 Curriculum learning design

Prior research demonstrates that curriculum learning—where task difficulty is incrementally increased—is a highly effective strategy for training complex locomotion policies. Accordingly, we propose an adaptive terrain curriculum designed to facilitate effective learning for bipedal wheel-legged robots in unstructured environments. At the onset of training, robots are uniformly distributed across the lowest difficulty tier of each terrain type, and the terrain difficulty level is dynamically updated based on the robot’s real-time performance. If the robot successfully traverses more than half the terrain length within a fixed time, it is deemed to have mastered the current difficulty. Consequently, the terrain level is incremented upon the next reset. Conversely, the difficulty level is decremented, allowing the robot to consolidate basic skills on simpler terrain. To mitigate catastrophic forgetting, robots that reach the maximum difficulty level are randomly reassigned to intermediate terrains.

## 3 Result

### 3.1 Training performance

**FIGURE 3:**
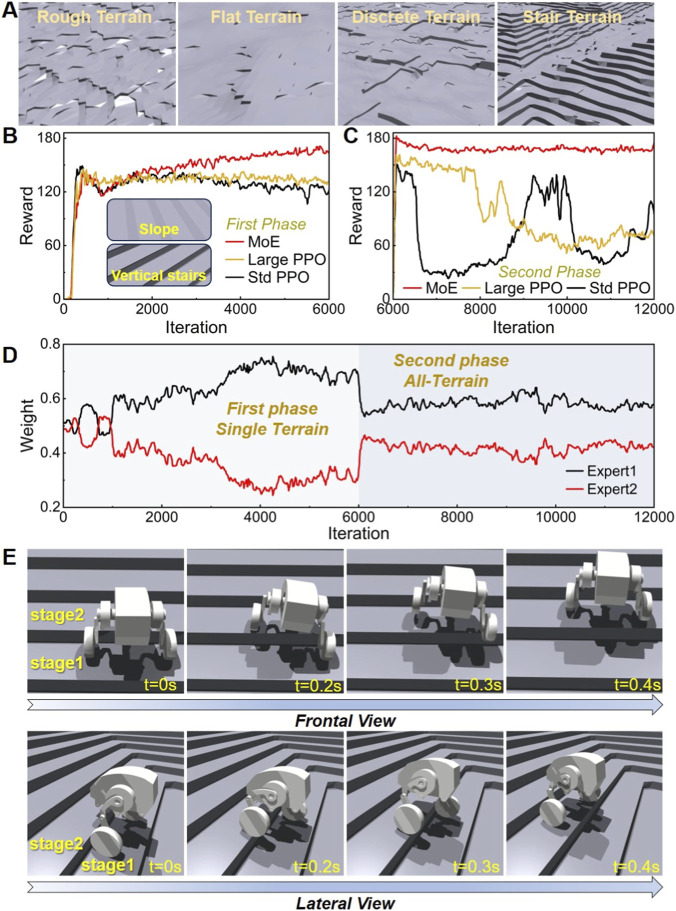
Terrain environment and stair climbing tests. **(A)** The training terrain after the addition of Perlin noise. **(B)** Comparison of training reward curves among the standard PPO baseline, large PPO baseline, and MoE in the first training phase. **(C)** Comparison of training reward curves among the standard PPO baseline, large PPO baseline, and MoE in the second training phase. **(D)** Average weight changes of the two expert networks throughout the training process. **(E)** Results of the robot using the trained locomotion policy to ascend stairs, with action decompositions shown from both frontal and lateral views.

Since the Actor in MoE-enhanced algorithm contains two networks of size [512, 256, 128], its total parameter count is approximately twice that of the standard PPO baseline. To exclude the impact of increased network capacity, this study introduces an additional large PPO baseline with a similar parameter count to MoE-enhanced algorithm. The large PPO baseline has a network size of [768, 384, 192], corresponding to scaling each layer of the Actor in the standard PPO baseline by a factor of 1.5. In both training phases, all three algorithms are trained with identical hyperparameters, including reward term weights, terrain settings, randomization parameters, and total training iterations. In the first phase, no Perlin noise is applied to the terrain, while in the second phase, Perlin noise is added to introduce irregular surface disturbances, as shown in Figure 3A. Both phases contain 6,000 training iterations, and the average rewards are compared, with results presented in Figures 3B,C, respectively. In the first phase, no obvious training collapse is observed for the three algorithms. Before around 1,600 iterations, their reward values and convergence speeds are nearly identical. After 1,600 iterations, the reward curves diverge significantly: the reward of the MoE-enhanced algorithm rises steadily, while that of the standard PPO baseline declines continuously. Although the reward of the large PPO baseline does not drop obviously, it shows no upward trend. By the end of the first phase, the reward of the MoE-enhanced algorithm is significantly higher than the other two baselines. Initially, the terrain mainly includes flat ground, slopes, and low stairs with small differences in dynamic properties. For low stairs, the robot can even traverse them via pure rolling, with dynamics similar to normal flat-ground locomotion and slope traversal. As the training progressed and stair heights increased, reaching a maximum height of 14 cm, which is much larger than the wheel radius, the robot can only overcome such obstacles through jumping or leg-lifting stepping, whose dynamics differ greatly from pure rolling. Training with a single network easily causes gradient update interference: after learning leg-lifting or jumping, the robot partially forgets pure rolling; similarly, improving pure rolling leads to partial forgetting of obstacle-crossing skills. Thus, the policy learned by a single network is a compromised solution. When facing higher stairs, its performance is insufficient, leading to stagnant or even severely degraded rewards.

In the second phase, Perlin noise with an amplitude of 6 cm is introduced into the terrain, and terrain types are extended to full terrain. Under these conditions, both the standard PPO baseline and large PPO baseline exhibit severe training instability: reward curves fail to converge, and training collapse frequently occurs. The primary cause is that Perlin noise increases terrain surface unevenness, leading to large fluctuations in the robot’s state observations at each time step. The policy continuously chases changing optimal behavior, resulting in reward volatility. If the frequency of terrain variations exceeds the adaptation speed of the policy, training collapse occurs. For single-network algorithms, limited learning capacity causes a trade-off dilemma, making stable training infeasible on rough terrain and preventing a robust final policy. In contrast, the MoE-enhanced algorithm maintains favorable reward curves throughout entire training process. In the first phase, its policy gradually adapts to increasing stair heights, with consistent reward rises. In the second phase, since the two expert networks’ skills were specialized in the first phase, with each expert responsible for distinct gait tasks, stable locomotion can be achieved by alternating between different experts even on rough terrain surfaces, thereby ensuring training stability. To further verify MoE-enhanced algorithm stability, we trained all three algorithms with different random seeds. The results are shown in Supplementary Figure S1 of the Supplementary Material. The results demonstrate that the MoE-enhanced algorithm performs stably across different seeds, whereas the single-network standard PPO and large PPO baselines exhibit significant variations across seeds and struggle to learn a stable policy. Overall, these results preliminarily validate the inherent limitations of single-network structures when handling multi-mode locomotion tasks in complex environments. The performance improvement of the MoE-enhanced algorithm arises primarily from its intrinsic mechanism, rather than the increase in total network parameters.

### 3.2 Experts contributions during training

The variation in the average weights of the experts during the training process of the MoE-enhanced algorithm was extracted in Figure 3D. It is evident that in the first phase, there was a substantial difference in the call frequencies between the two expert networks; however, this disparity significantly decreased in the second phase. The primary reason for this change can be attributed to the relatively simple terrain in the first phase, which consisted solely of flat surfaces, slopes, and vertical stairs without any added Perlin noise. In this scenario, the use of the leg-lifting gait was required only for navigating the vertical stairs. When encountering vertical stairs, the robot needed to roll a certain distance on the step before transitioning to the next one, which meant that calls to the expert responsible for the leg-lifting gait were infrequent, occurring only during the transition from one stair to the next, while the majority of the time involved pure rolling. Consequently, during the first phase of training, the call frequency for the leg-lifting expert (Expert 2 in the figure) was significantly lower than that of the expert responsible for pure rolling (Expert 1), leading to a notable difference in their average weights as the number of training epochs increased. In contrast, the second phase introduced discrete terrains that required frequent alternation between pure rolling and the leg-lifting gait, along with the addition of Perlin noise, which exacerbated the irregularity of the terrain surface. This heightened the demand for the leg-lifting gait. Under these terrain conditions, the call frequency for Expert 2 in the second phase noticeably increased to accommodate the undulating ground. As the number of training epochs in the second phase increased, the difference in average weights between the two experts significantly diminished.

### 3.3 Stair-climbing test

The locomotion policy derived from the MoE-enhanced algorithm was tested for ascending stairs, with the results presented in Figure 3E. When faced with stairs of a vertical height of 12 cm, the robot successfully executed an ideal leg-lifting gait. The motion breakdown was analyzed from both the frontal and lateral views, as shown in Figure 3E. With a speed command set at 0.6 m/s, the robot took approximately 0.4 s to clear the step. During the ascent, the robot first lifted its left wheel onto the next step before bringing up the right wheel, thereby effectively utilizing the leg-lifting gait to complete the stair-climbing task. In general, for bipedal robots encountering vertical obstacles, common motion policies include jumping or employing a leg-lifting gait. However, in this study, due to the incorporation of a significant leg-lifting reward, the resulting obstacle negotiation policy was exclusively the leg-lifting gait. Utilizing only the leg-lifting gait for obstacle negotiation effectively addresses several issues associated with jumping, such as problems related to body stability and the impacts and vibrations that may affect the robot’s internal components.

### 3.4 Obstacle navigation test

**FIGURE 4:**
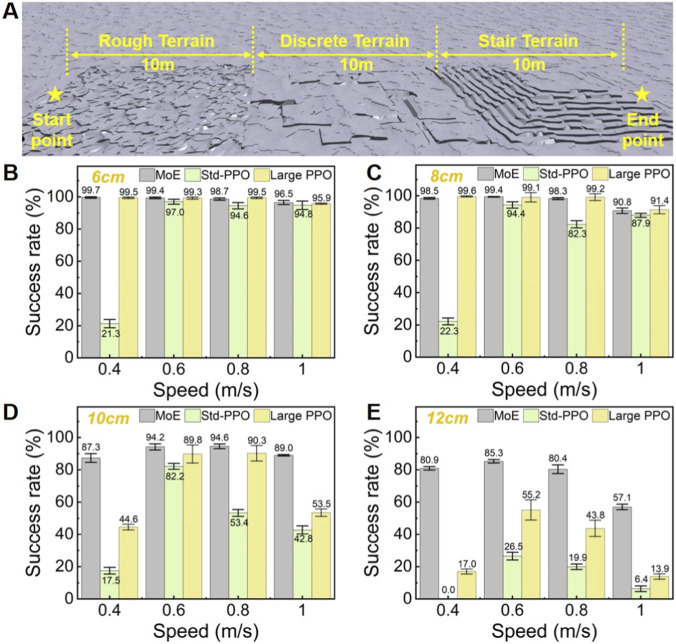
Obstacle navigation test. Success is defined as the robot traveling from the “Start point” to the “End point” without colliding with the ground and without straying outside the 10-m-wide test area. For each test statistic, 500 robots are tested independently, and the final success rate of these 500 robots is calculated. **(A)** Setup of the obstacle testing area. **(B)** Comparison of success rates for the three algorithms at a maximum vertical height of 6 cm. **(C)** Comparison of success rates for the three algorithms at a maximum vertical height of 8 cm. **(D)** Comparison of success rates for the three algorithms at a maximum vertical height of 10 cm. **(E)** Comparison of success rates for the three algorithms at a maximum vertical height of 12 cm.

A specially designed testing environment is established to test the obstacle negotiation success rates of the two policies (Figure 4A), which included three types of terrain: rough terrain, discrete terrain, and vertical stairs. Each terrain type was set within a square area, with the rough terrain generated using Perlin noise at an amplitude of 6 cm, and the maximum vertical heights for the discrete terrain and vertical stairs set between 6 and 12 cm. During the testing process, a successful trial required the robot to remain upright throughout, not exit the terrain boundaries, and reach the endpoint. Each terrain with distinct difficulty was measured repeatedly, and 500 robots were used in each single test for statistical analysis. The results are shown in Figures 4B,C. From the results, the locomotion policy trained by the MoE-enhanced algorithm significantly outperforms the standard PPO baseline and the large PPO baseline. In terrains of all difficulty levels, its success rate exceeds 50% across all velocity commands. For the standard PPO baseline, insufficient obstacle-crossing performance can be clearly observed, especially under low velocity commands. Even in the simplest terrain with an obstacle height of 6 cm, its success rate remains low. In the terrain with a height of 12 cm, it performs poorly across all velocity commands. The large PPO baseline also exhibits a considerable performance gap compared with the MoE-enhanced algorithm, and this gap becomes increasingly pronounced as terrain difficulty rises. In particular, when the maximum vertical obstacle height reaches 12 cm, the success rate of the large PPO baseline is generally below 50%. Overall, these results further demonstrate that the superiority of the MoE-enhanced algorithm mainly stems from the inherent mechanism of MoE, and is not significantly affected by the specific amount of network parameters.

Based on this experiment, we further conducted ablation experiments to analyze the effects of MoE and phased training. We established an additional baseline trained directly for 12,000 iterations without phasing. Similarly, we evaluate the two training schemes using two metrics: the average training reward and the obstacle-crossing success rate on terrains of different heights. First, with respect to the average reward, training was performed with four different random seeds, and the reward results were statistically analyzed, as shown in Supplementary Figure S2 in the Supplementary Material. It can be clearly observed that the MoE-enhanced algorithm achieves higher reward values under phased training than under non-phased training. Subsequently, the locomotion policies obtained by the two training methods were tested for obstacle-crossing success rate, with the results presented in Supplementary Figure S3 in the Supplementary Material. The results show that the performance difference between the two policies is not significant on relatively simple terrains. When the maximum vertical height of obstacles reaches 12 cm, the success rate of the policy trained with phased training is significantly higher than that of the non-phased policy. This validates that phased training can improve the upper bound of obstacle-crossing performance. Combined with the comparison results against the PPO algorithm using a single network, it can be concluded that in the MoE-enhanced algorithm proposed in this study, the mechanism of MoE itself plays a more dominant role, while phased training serves as an auxiliary approach to further improve the algorithm’s performance.

## 4 Conclusion

This study proposes a reinforcement learning control framework that integrates the Mixture of Experts (MoE) for bipedal wheel-legged robots. By incorporating MoE into the Actor network of the PPO algorithm, we leverage a sparse gating mechanism to decouple parameters for pure rolling and leg-lifting tasks, effectively resolving the gradient conflict issues associated with single-network multi-task learning. Simulation test results demonstrate that this approach not only eliminates catastrophic forgetting but also significantly enhances training stability. To ensure the robustness and generalization capabilities of the policy, we implemented a two-phase curriculum learning strategy that transitions from specific terrains to complex terrains with Perlin noise. The MoE-enhanced strategy exhibits remarkable adaptability when navigating complex terrains, automatically adjusting expert weights according to the terrain’s undulations, thus achieving a smooth transition from rolling on flat ground to dynamic obstacle negotiation. Notably, in high-difficulty obstacle tests, this policy achieved a success rate that greatly exceeded that of the single-network PPO algorithm when traversing vertical stairs of 12 cm. Future work will focus on several key areas: recognizing the significant impact of collision forces on balance during high-speed movements, further research will aim to enhance the system’s active safety and self-recovery capabilities under extreme physical contact and external disturbances. Additionally, we will conduct real-world deployment tests in more diverse and unstructured outdoor environments to validate and optimize the long-term reliability of this method in practical applications.

## Data Availability

The raw data supporting the conclusions of this article will be made available by the authors, without undue reservation.
