# Supplementary material for: Adaptive multi-mode locomotion for bipedal wheel-legged robots via sparse mixture-of-experts deep reinforcement learning
Source: Front Robot AI. 2026 Feb 25;13:1788395. doi: 10.3389/frobt.2026.1788395 (PMC12975443; doi:10.3389/frobt.2026.1788395)
Supplement: Supplementary file 1 [file DataSheet1.pdf]

Supplementary Material

Adaptive multi-mode locomotion for bipedal wheel-legged robots via sparse mixture-of-experts deep reinforcement learning

Pan He[1], Zeang Zhao[1]*, Shengyu Duan[1]*, Panding Wang[1], Hongshuai Lei[1]

Affiliations

[1]Institute of Advanced Structure Technology, Beijing Institute of Technology, Beijing 100081, China

*Corresponding author: Email: zza@pku.edu.cn (Z.Z);

Email: shengyu_duan@126.com (S.D).

This PDF file includes:

Text S1

Pseudocode S1 to S2

Figs. S1 to S4

Tables S1 to S4

1 Supplementary Text

Text S1: Sensitivity analysis of the leg-lifting reward coefficient

Since the leg-lifting reward term is a core reward in this study, we performed a sensitivity analysis on this reward term. We set different leg-lifting reward coefficients and tested the obstacle-crossing success rate of the locomotion policies trained under these coefficients. The results are shown in Supplementary Figure S4. From the results, the performance of the trained algorithm is optimal when the leg-lifting reward coefficient is set to 20. When the coefficient is reduced to 15, the performance drops sharply, and the robot almost loses its obstacle-crossing capability. When the reward coefficient is increased to 25 or 30, the performance also degrades significantly. In particular, with a coefficient of 30, the obstacle-crossing success rate approaches zero for obstacles with a height of 12cm. When the reward coefficient is too small, the incentive for learning leg-lifting motions is insufficient. The reward from leg lifting cannot compensate for the penalties caused by higher joint torques and collision risks, so the policy tends to avoid leg lifting during training. In contrast, when the leg-lifting reward is excessively high, the policy overemphasizes leg-lifting actions to pursue high rewards. During the early training stage, frequent leg lifting introduces severe instability and safety hazards, making the robot prone to falling and receiving large penalties (−180). Such penalties further discourage the policy from performing leg-lifting motions, which suppresses learning from the beginning and results in poor obstacle-crossing performance. In summary, the leg-lifting reward coefficient has an appropriate range: values that are either too small or too large cannot produce an effective locomotion policy. A value of 20 is reasonable in this study, so the policy trained with this coefficient is used for all subsequent experiments and analysis.

2 Supplementary Algorithm Pseudocode

Pseudocode S1: The baseline PPO training loop

1: Initialize policy network π_θ , value network V_ψ , and replay buffer D
2: Set learning rate α , clipping parameter ε , discount factor γ , and GAE parameter λ
3: **For** iteration $m = 1, 2, ..., M$ **do**:
4:     **For** time step $t = 1, 2, ..., T$ **do**:
5:         Observe state S_t , sample action a_t
6:         Execute a_t in Isaac Gym, store transition (S_t , a_t , r_t , S_{t+1}) in D
7:     **End for**
8:     Compute Generalized Advantage Estimator (GAE) $\hat{A}_t$ and rewards-to-go R_t
9:     **For** epoch $k = 1, 2, ..., K$ **do**:
10:         Sample mini-batch $B \subset D$
11:         Compute Clipping Loss: $L_{clip} = E_B[\min(\rho_t \hat{A}_t, clip(\rho_t, 1-\varepsilon, 1+\varepsilon)\hat{A}_t)]$
12:         Compute Value Loss: $L_{vf} = E_B[(V_\psi(S_t) - R_t)^2]$
13:         Update θ and ψ by minimizing $L_{total} = -L_{clip} + c_1 L_{vf} - c_2 H$
14:     **End for**
15:     Clear replay buffer D and repeat
16: **End for**

Pseudocode S2: MoE Control and Load Balancing

```
Receive current robot state S_t (proprioceptive and historical data)
Pass S_t through Gating Network G_ψ to get raw scores H(S_t) = G_ψ(S_t)
Feed S_t into each expert network to generate actions a_1, a_2, ..., a_n
Calculate gating weights W = Softmax(H(S_t))
Apply Top-K Selection: keep the largest weight and set others to zero
Output the max-weight expert's action a_t
Compute load balancing loss L_aux from the average weight of each expert
Scale L_aux and add to the total PPO optimization loss.
```

3 Supplementary Figures and Tables

3.1 Supplementary Figures

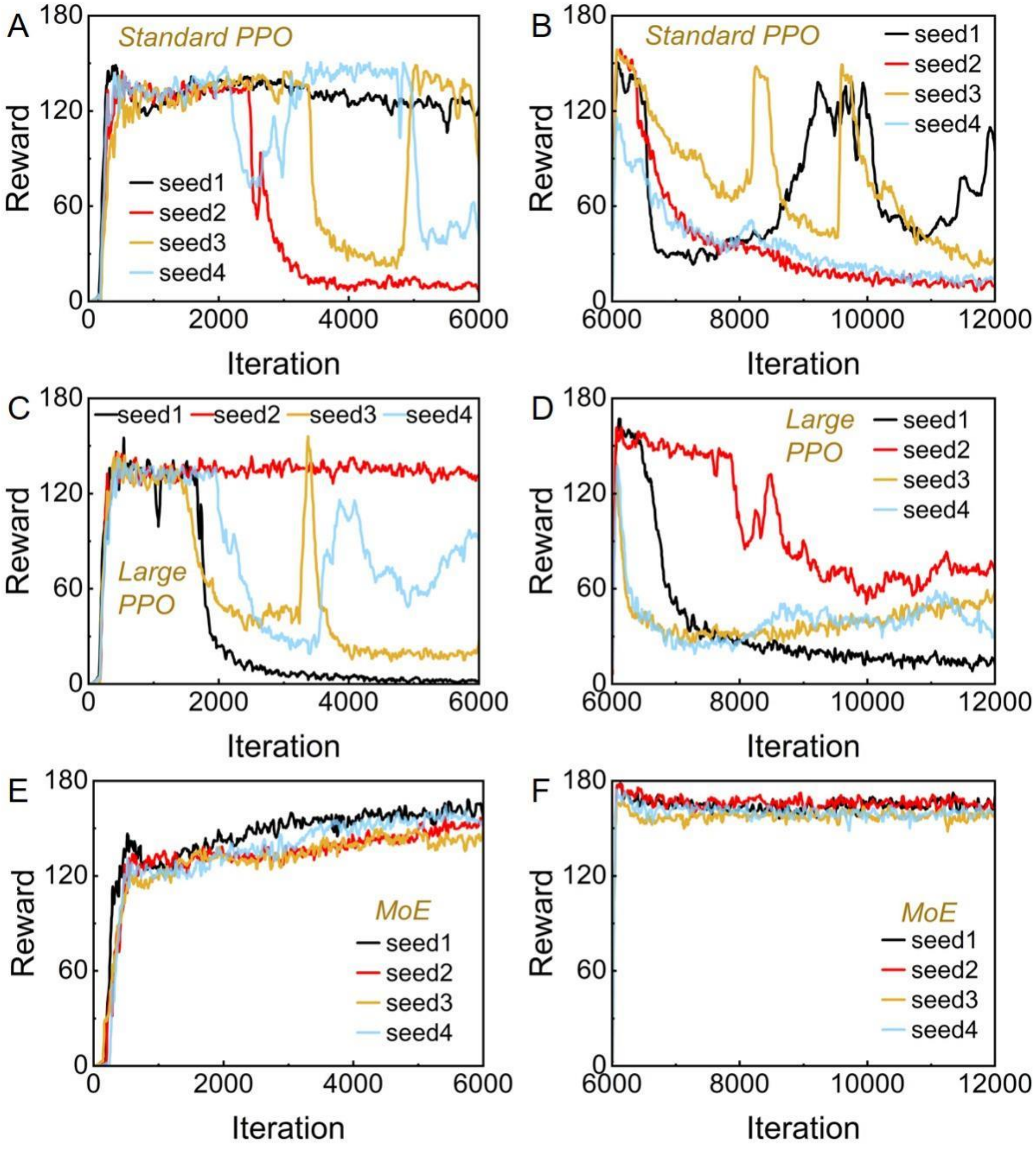

Supplementary Figure S1. Training reward curves of the three algorithms in this study across different random seeds. The three algorithms proposed in this study are trained with four distinct random seeds. The results indicate that the variation of random seeds has little influence on the training stability of the MoE-enhanced algorithm, whereas the training results of the standard PPO baseline and the large PPO baseline vary significantly with different seeds. **(A)** Training reward curves of the standard PPO baseline in the first phase. **(B)** Training reward curves of the standard PPO baseline in the second phase. **(C)** Training reward curves of the large PPO baseline in the first phase. **(D)** Training reward curves of the large PPO baseline in the second phase. **(E)** Training

reward curves of the MoE-enhanced algorithm in the first phase. **(F)** Training reward curves of the MoE-enhanced algorithm in the second phase.

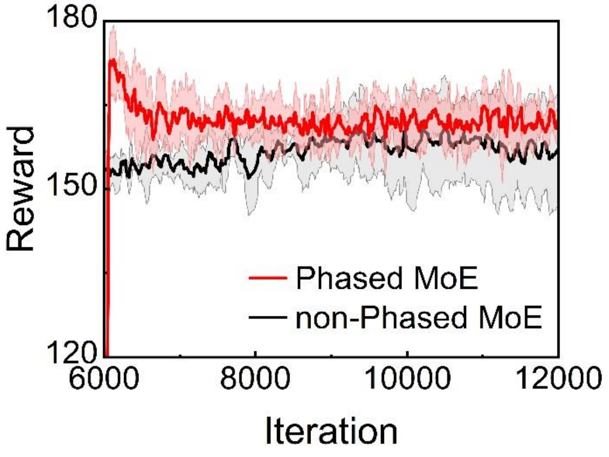

Supplementary Figure S2. Comparison of training rewards between phased and non-phased training of the MoE-enhanced algorithm on Perlin noise terrain. Since the terrain in the first phase of the phased training setup contains no Perlin noise, this study only compares and analyzes the training reward results from the last 6000 iterations.

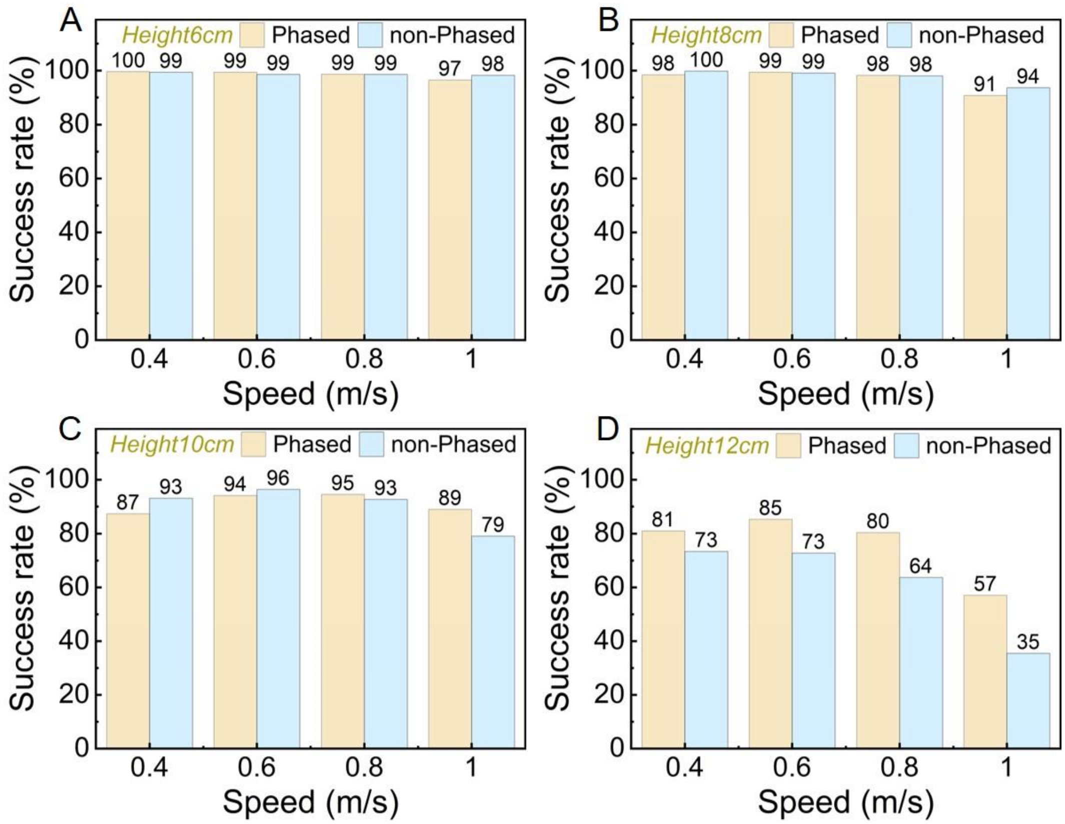

Supplementary Figure S3. Comparison of average obstacle-crossing success rates between policies obtained by phased and non-phased training of the MoE-enhanced algorithm. (A) Average obstacle-crossing success rates on terrain with a maximum vertical height of 6 cm. **(B)** Average obstacle-crossing success rates on terrain with a maximum vertical height of 8 cm. **(C)**

Average obstacle-crossing success rates on terrain with a maximum vertical height of 10 cm. **(D)** Average obstacle-crossing success rates on terrain with a maximum vertical height of 12 cm.

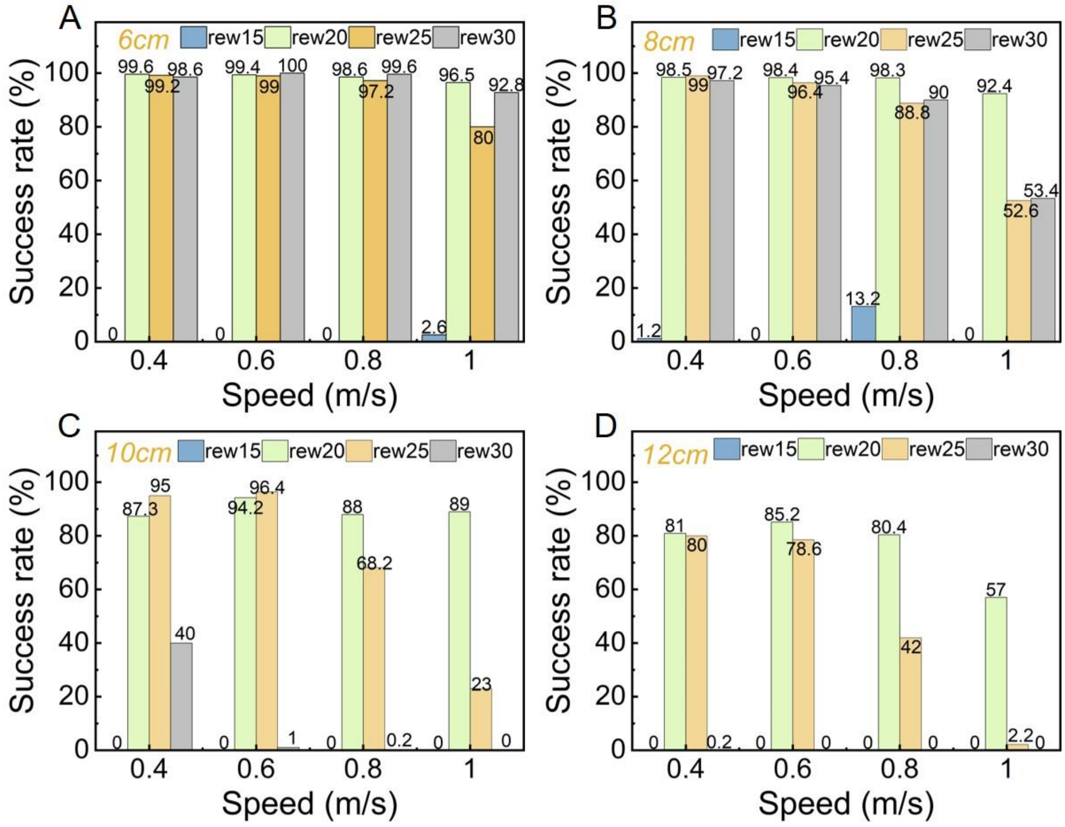

Supplementary Figure S4. Comparison of average obstacle-crossing success rates of the MoE-enhanced algorithm under different leg-lifting reward coefficients. (A) Comparison of obstacle-crossing success rates on 6 cm height terrain. **(B)** Comparison of obstacle-crossing success rates on 8 cm height terrain. **(C)** Comparison of obstacle-crossing success rates on 10 cm height terrain. **(D)** Comparison of obstacle-crossing success rates on 12 cm height terrain.

3.2 Supplementary Tables

Supplementary Table S1. Hyperparameters of the baseline PPO algorithm

Hyperparameter	Value	Description
Learning rate	0.001	Step size for updates
Clip parameter	0.2	PPO surrogate loss clipping range
Discount factor	0.99	Importance of future rewards
GAE parameter	0.95	Bias-variance tradeoff for advantage
Entropy coefficient	0.01	Encourages exploration
Mini-batch size	4	Number of subsets per epoch

Num learning epochs	5	Number of updates per batch
Desired KL	0.01	Targeted KL divergence for adaptive learning rate
Linear Velocity(x)	[-1, 1]	Forward linear velocity command (m/s)
Linear Velocity(y)	[0, 0]	Lateral linear velocity command (m/s)
Angular Velocity(yaw)	[-1, 1]	Yaw rate command (rad/s)
Friction Range	[0.5,1.25]	Ground friction coefficient randomization
Resampling time	10	Interval to sample new commands (s)
Added Mass Range	[-1, 1]	Payload mass perturbation (kg)
Actor Hidden Dims	[512,256,128]	Dimension of the Actor network
Critic Hidden Dims	[512,256,128]	Dimension of the Critic network

Supplementary Table S2. Hyperparameters of the MoE-enhanced algorithm

**Hyperparameter**	**Value**	**Description**
Number of Experts	2	Total number of expert networks
Top-k Selection (k)	1	Number of activated experts per time step
Load Balance Coef	0.001	For load balancing loss regulation
Expert Hidden Dims	[512,256,128]	Dimension of the expert network
Gating Network Dims	[128,64]	Dimension of the gating network

Supplementary Table S3. Hyperparameters of the PD controller

**Hyperparameter**	**Value**	**Description**
Proportional Gain K_p	80	Stiffness of the virtual spring at each joint
Derivative Gain K_d	5	Damping coefficient to suppress joint oscillations
Action Scale	0.25	Scaling factor for the neural network output
Decimation	4	Number of simulator steps per policy step

Supplementary Table S4. Table of noise amplitudes in the observation space. In this study, different noise levels are applied to distinct raw sensor data.

**Observation**	**Value**	**Description**
Dof pos	0.01	Joint position sensing noise (rad)
Dof vel	1.5	Joint velocity sensing noise (rad/s)
Lin vel	0.1	Base linear velocity noise (m/s)
Ang vel	0.2	Base angular velocity noise (rad/s)

Gravity	0.05	Gravity vector noise (m/s^2)
